# Cinnamic Acid Toxicity on the Structural Resistance and Photosynthetic Physiology of Faba Bean Promoted the Occurrence of Fusarium Wilt of Faba Bean, Which Was Alleviated Through Wheat and Faba Bean Intercropping

**DOI:** 10.3389/fpls.2022.857780

**Published:** 2022-06-08

**Authors:** Wenhao Yang, Yuting Guo, Yu Li, Yiran Zheng, Kun Dong, Yan Dong

**Affiliations:** ^1^College of Resources and Environment, Yunnan Agricultural University, Kunming, China; ^2^College of Animal Science and Technology, Yunnan Agricultural University, Kunming, China

**Keywords:** cinnamic acid, faba bean, fusarium wilt, toxic action, defense capability, photosynthetic characteristics, intercropping

## Abstract

**Background:**

The pattern of intercropping wheat and faba bean is an effective means to alleviate continuous cropping obstacles.

**Aim:**

To study the mechanism by which cinnamic acid promotes faba bean wilt and the mechanism by which intercropping alleviates this effect.

**Methods:**

Hydroponics was used to study the effects of inoculation with or without *Fusarium oxysporum* f. sp. *fabae* (FOF) and the effect of addition of different concentrations of cinnamic acid on seedling growth, Fusarium wilt, stem cell wall degrading enzyme activity, lignin content, tissue structure of the stem and leaf photosynthesis in monocropping and intercropping systems following the inoculation of faba bean with FOF.

**Results:**

Treatment with FOF significantly reduced the biomass and leaf photosynthesis of faba bean compared with the control. Microscopic observation showed that the xylem vessels of the stem were slightly thickened. Compared with FOF alone, the combination of FOF and cinnamic acid stress significantly increased the activity of cell wall degrading enzymes (CWDEs) produced by FOF in the stem and content of lignin in the stem. Microstructural observation showed that cell wall thickening of the xylem conduit, stratification, formation of a cavity and even caused the dispersion of tissue cell structure in the stem tissue of faba bean. Furthermore, the biomass and leaf photosynthesis of faba bean decreased significantly, and the occurrence of faba bean wilt increased. Compared with the faba bean monocropping treatment, the wheat and faba bean intercropping treatment significantly reduced the activity of CWDEs of FOF produced in faba bean stems and increased the lignin content. In addition, observation of the microstructure indicated that the tissue structural cell wall thickened after the stem had decreased, and the amount of colloidal substances and their containment decreased, causing a further decrease in tissue deformation, smaller intercellular spaces, less divided layer cell damage, an increase in the aboveground biomass and leaf photosynthesis of faba bean and a decrease in the occurrence of faba bean wilt.

**Conclusion:**

Cinnamic acid decreased the resistance of tissue structure and promoted the occurrence of wilt. Wheat and faba bean intercropping improved the resistance of tissue structure, which reduced the occurrence of wilt.

## Introduction

As theworld’s population continues to grow, the demand for food and cash crops also increases. Owing to the limitations in arable land, the area of arable land being added is reduced, and the continuous planting of the same crops on the same land has become the most common mode in intensive and large-scale agriculture and horticultural production (Alexandratos and Bruinsma, 2012; Zeng et al., 2020). However, continuous planting for many years can lead to continuous cropping disorders. Continuous cropping obstacles can cause weak plant growth, a reduction in yield, poor quality and an increase in soilborne diseases (Li et al., 2016; Gao et al., 2019; Zeng et al., 2020). Among them, the frequent occurrence of soilborne diseases has always been an intractable problem in actual production (Young, 1984; Grodzinsky, 1992). The root cause of soilborne diseases is that the number of soilborne pathogens exceeds a critical value (Elmstrom and Hopkins, 1981; Caperton et al., 1986). Most soilborne diseases are common in soybean (*Glycine max*) (Dhingra and Muchovej, 1979; Haddoudi et al., 2020), potato (*Solanum tuberosum*) (Rai, 1979), cowpea (*Vigna unguiculata*) (Aigbe et al., 1999), tomato (*Solanum lycopersicum*) (Nedumaran and Vidhyasekaran, 1981) and other field crops and cash crops. Soilborne pathogenic fungi can survive for several years or even decades in the soil in the absence of a host (Buruchara and Camacho, 2000; De Borbat et al., 2017). The number of soilborne pathogens is regulated by allelopathy (Wu et al., 2008a).

Allelopathy is the inhibition or promotion of chemicals, which are released into the environment by one plant to affect another (Rice, 1984). Autotoxicity is a special form of allelopathy in which plants produce toxic substances primarily through root secretion or the decomposition of residual roots, thereby inhibiting their own growth (Singh et al., 1999). Li et al. (2018) found that autotoxic substances were released into the rhizosphere soil of ginseng (*Panax ginseng*) and accumulated to some concentration, thus, inhibiting its growth. In recent years, increasing amounts of attention have been paid to allelopathic autotoxicity, which plays a key role in the occurrence of soilborne diseases (Huang et al., 2013; Li et al., 2014a; Zhang et al., 2020). Studies have shown that cinnamic acid secreted by asparagus (*Asparagus officinalis*) is considered as the primary toxin of asparagus roots, which can stimulate *Fusarium* spp. to infect asparagus and promote soilborne diseases (Peirce and Miller, 1993). The accumulation of autotoxic substances in flower rhizospheres aggravates the occurrence of soilborne peanut diseases (Li et al., 2013). Wu et al. (2008b) found that the activities of pectinase, cellulase, amylase and protease produced by *F. oxysporum* increased significantly after cinnamic acid was added to the culture medium of this fungus. The CWDEs can degrade the host tissue structure (Klechkovskaya et al., 1998; Pekkarinen et al., 2000; Yi and Wu, 2000; Aparna et al., 2009). The results showed that the autotoxic substances promoted the production of CWDEs by pathogens, which is an important way to promote the occurrence of soilborne diseases. It has also been suggested that allelopathic autotoxic substances promote disease occurrence by reducing the resistance of crops to pathogens (Nighjr, 1990). Under the stress of *p*-hydroxybenzoic acid, the degree of damage to strawberry root tissue structures was aggravated, which significantly increased the infection rate of *F. oxysporum* and promoted the occurrence of wilt (Qi et al., 2015). It has been reported that under cinnamic acid stress, the stomatal conductance and net photosynthetic rate of cucumber (*Cucumis sativa*) leaves decreased, which inhibited the photosynthesis of the leaves and further promoted the occurrence of cucumber Fusarium wilt (Ye et al., 2004). The results showed that the autotoxic compounds reduced the resistance of plants to pathogens by reducing the resistance of their tissues, cells, and photosynthesis, rendering the plants more susceptible to infection and increasing the incidence of diseases.

Currently, the prevention and control of soilborne diseases in agricultural production generally comprises physical prevention and control, chemical prevention and control, and other methods. Steam high-temperature disinfection of soil is a simple and effective method to prevent soilborne diseases (Katan, 1980). However, the disinfection of soil with steam easily causes secondary colonization and the mass enrichment of pathogenic microorganisms, resulting in negative effects on the subsequent growth of crops (Fenoglio et al., 2006). Chemical prevention and control primarily use various chemical agents to control soil pathogens. Mao et al. (2012) fumigated a cucumber nursery with 98% methyl isothiocyanate, which greatly reduced the number of *F. oxysporum* propagules in soil. However, these chemical methods not only eliminate pathogens in the soil but also kill beneficial microorganisms, aggravate environmental pollution and disrupt the soil microecological balance. All these control methods have their limitations. Therefore, the development of effective and environmentally friendly soilborne disease management strategies has been a key research focus (Chen et al., 2019). Breeding resistant varieties is considered the most direct and effective measure to combat wilt. Studies have shown that the root exudates of resistant peanut (*Arachis hypogaea*) species (“quanhua-7”) significantly reduced the number of spores and amount of spore germination compared with non-resistant peanut species (“guanhua-5”) (Li et al., 2013). This could be an important route for disease-resistant varieties to inhibit the occurrence of wilt. Simultaneously, reasonable intercropping is a method of planting two or more crops together. In practical production, it is a green and efficient planting method and is often used to control soilborne diseases (Li et al., 2014b; Ren et al., 2016). Studies have shown that compared with pea and corn monocropping, intercropping of corn and pea increased the yield of pea and promoted the growth of corn (Hu et al., 2016). It has been reported that maize (*Zea mays*) and potato intercropping can effectively control the occurrence of potato Fusarium wilt (Autrique and Potts, 1987). Intercropping of *Atractylodes lancea w*ith peanut can reduce the incidence of root rot of continuously cropped peanut (Li et al., 2014b). The results showed that intercropping could effectively promote crop growth and inhibit the occurrence of Fusarium wilt. Currently, the mechanism of intercropping to alleviate soilborne diseases primarily focuses on the effects of intercropping on pathogen growth, rhizosphere microflora and community structure. It has been reported that the incidence of watermelon *F. oxysporum* in paddy and watermelon intercropping in dry farming decreased by decreasing the number of watermelon *F. oxysporum* and rhizosphere fungi and increasing the number of soil bacteria in the root zone (Ren et al., 2008). Intercropping with onion (*Allium cepa*), garlic (*A. sativum*) and cucumber changed the microbial community structure of cucumber soil and reduced the incidence of cucumber Fusarium wilt (Xiao et al., 2012). The results showed that intercropping could reduce the incidence of soilborne diseases by reducing the number of pathogens and improving the rhizosphere microflora and community structure. However, there have been few studies on how intercropping regulates the effects of pathogenic factors, such as CWDEs, tissue structure resistance and photosynthetic physiology in plants.

As one of the oldest crops in the world, faba bean (*Vicia faba* L.) is widely cultivated all over the world, providing a large amount of protein for humans and animals, and is valuable for medicine and health care (El Idrissi et al., 2020). However, continuous cultivation of faba beans frequently results in soilborne wilt (Stoddard et al., 2010). In Yunnan Province of southwest China, wheat is often grown with faba beans to control the bean wilt. Few studies have explored the mechanism of the occurrence of faba bean wilt owing to the synergistic effect of *F. oxysporum* and autotoxic substances and mitigation of this disease owing to the effect of intercropping systems. Our previous study showed that cinnamic acid is one of the primary autotoxic substances of faba bean, and its pathogenic mechanism has been studied from the ability of cinnamic acid to help faba bean become resistant to *Fusarium oxysporum* f. sp. *fabae* (FOF) and produce defense enzymes (Guo et al., 2020). However, the synergistic effects of FOF and cinnamic acid on pathogenicity and the resistance of faba bean tissue structure, as well as the alleviating mechanism of the wheat and faba bean intercropping system, are still unclear. Therefore, this study utilized a hydroponics experiment to determine the following: (1) the synergistic effect of FOF and cinnamic acid on the occurrence of faba bean wilt and the ability of wheat intercropping to mitigate the infection; and (2) the synergistic effect of FOF and cinnamic acid on promoting the occurrence of faba bean wilt and the potential mechanism of wheat and faba bean intercropping to effectively control the occurrence of faba bean wilt by reducing its pathogenicity and enhancing the resistance of faba bean tissue structure.

## Materials and Methods

### Test Materials

Faba bean seeds of the resistant disease variety “89–147” and wheat variety “Yunmai 53” were purchased from the Yunnan Academy of Agricultural Sciences (Kunming, China) (Fabae Yu et Fang, FOF; *Fusarium oxysporum* Schlecht. f. sp *fabae* Yu et Fang) was isolated from an infected, continuously cropped faba bean plot. The spores were collected by filtration with four layers of gauze and diluted into a suspension (≤ 1 × 10^6^ CFU⋅mL^–1^) for plant inoculation. The spores were cultured on potato dextrose agar (PDA) plates and incubated at 28°C for 7 days at a constant temperature.

A volume of 90 L of Hoagland Nutrient Solution configuration. The mass elements CaCl_2_⋅6H_2_O 135 g, KNO_3_ 45.9 g, MgSO_4_⋅7H_2_O 44.1 g, KH_2_PO_4_ 12.6 g and trace elements H_3_BO_3_ 257.4 g, MnCl_2_⋅4H_2_O 162.9 g, ZnSO_4_⋅7H_2_O 19.8 g, CuSO_4_⋅5H_2_O 7.2 g, (NH_4_)6Mo_7_O_24_⋅4H_2_O 8.1 g were mixed in 50 L of distilled water in a 100 L plastic bucket. A total of 501.3 g of FeSO4⋅7H_2_O was added to 7.2 L of distilled water and then boiled at 95°C in a water bath. A total of 670.5 g of Na_2_-EDTA was added and evenly stirred. Once cooled to room temperature, the solution was transferred to a 100 L plastic bucket. Finally, 32.8 L of distilled water was added to the plastic bucket.

### Experimental Design

The experiment was conducted in the glass greenhouse of Yunnan Agricultural University from September to December 2019. The experiment was conducted by hydroponics with nutrient solution in a multi-factor randomized design. Factor A was the inoculation treatment (without inoculation FOF: -F-0ca, inoculation FOF: +F+0ca). Factor B: four concentrations of cinnamic acid were added following the inoculation of FOF (inoculation FOF and 0 mg⋅L^–1^ cinnamic acid: +F+0ca, inoculation FOF and 50 mg⋅L^–1^ cinnamic acid: +F+50ca, inoculation FOF and 100 mg⋅L^–1^ cinnamic acid: +F+100ca, inoculation FOF and 200 mg⋅L^–1^ cinnamic acid: +F+200ca). Factor C was treated by two planting modes (faba bean monocropping: M, wheat and faba bean intercropping: I). Thus, the experiment had 10 treatments. Each treatment was conducted in triplicate, with each treatment and its replicates randomly allocated in the greenhouse, and the experiment was repeated three times.

**FIGURE 1:**
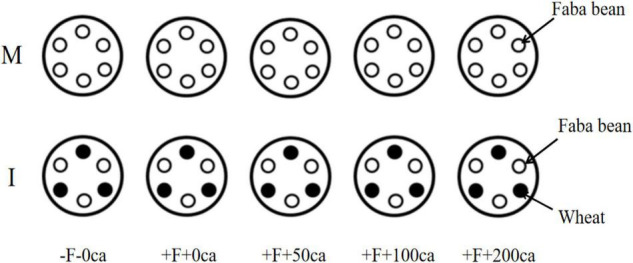
Schematic diagram of hydroponic cultivation of nutrient solution in faba bean monocropping and wheat and faba bean intercropping. Ca, cinnamic acid; F, *Fusarium oxysporum* f. sp. *fabae;* I, intercropping; M, monocropping.

A total of 300 full-sized uniform faba bean seeds and 200 full-sized uniform wheat seeds were treated in 10% (v/v) hydrogen peroxide (H_2_O_2_) for 30 min, germinated in the dark for 12 h in a saturated solution of CaSO_4_ and dark porcelain discs for 48 h. The germinated seeds were planted in sterile quartz sand soaked with water and watered daily at a set time. When the faba bean seedlings grew to 4∼6 true leaves and the wheat to three leaves, faba bean and wheat seedlings with the same amount of growth were selected and transferred into plastic basins (25 cm in upper diameter, 13 cm in lower diameter and 16 cm in height) filled with 2 L of Hoagland nutrient solution. Six faba bean seedlings were planted in each monoculture plastic basins (Figure 1). Simultaneously, three faba beans and three wheat plants were transplanted into each intercropping plastic basin (Figure 1). After 2 days of transplantation, 1 × 10^6^ CFU⋅mL^–1^ FOF spore suspension and different concentrations of cinnamic acid were added near the roots of faba bean based on different treatments. One plastic basin was used for each treatment. There were a total of 30 pots with 135 faba bean and 45 wheat plants. All the faba bean and wheat plants were grown under natural light, 26/19°C day/night temperatures, and 70–85% relative humidity. A ventilation pump was used for 24 h in the pots. The pH of the nutrient solutions ranged from 5.7 to 7.1 (Asaduzzaman and Asao, 2012). The nutrient solution was replaced every 2 days, and a ventilation pump was used continuously in the incubator.

### Measurement of Seedling Growth Parameters and Investigation of Faba Bean Wilt

Faba bean seedlings were transplanted for 45 days, and three faba bean plants with the same growth were selected from each treatment to measure the plant height and maximum leaf length and width. Each treatment was measured once and repeated three times independently.

The investigation of wilt was conducted 45 days after the transplantation of faba bean seedlings. In the faba bean monocropping treatment, one pot was investigated in each treatment; three faba beans were investigated in each pot, and each treatment was investigated once and repeated independently three times, with a total of nine plants. In the wheat and faba bean intercropping treatment, one pot was studied in each treatment; three faba bean plants were studied in each pot. Each treatment was investigated once and repeated independently three times, with a total of nine plants. The classification method of faba bean wilt was investigated using a 5-grade classification standard (Dong et al., 2016). Grade 0: asymptomatic; Grade 1: partial lesions or slight discoloration of the stem base or root (except for the primary root); Grade 2: diseased spots at the base of the stem or the main lateral root but not in patches; Grade 3: lesions, discoloration or decay appeared at one-third to one-half of the stem base or root, and the lateral roots were significantly reduced; Grade 4: the base of the stem was surrounded by disease spots, or most of the roots were discolored and rotten; Grade 5: the plants withered and died. The incidence and disease index were calculated as follows:


Incidence=⁢number⁢of⁢infected⁢plants/total⁢number⁢of⁢investigated⁢plants×100%

Disease⁢index=Σ⁢(number⁢of⁢diseased⁢plants⁢at⁢all⁢levels×corresponding⁢grade⁢value)/(highest⁢value×total⁢number⁢of⁢investigated⁢plants)×100%

### Preparation and Determination of the Activity of Cell Wall Degrading Enzymes From Faba Bean Stems

One gram of fresh stem was ground with in a mortar on ice, and the extract was centrifuged at 5,000 rpm for 30 min at 4°C to collect the crude enzyme solution. This solution was boiled for 10 min and centrifuged at 5,000 rpm for 30 min to collect the supernatant as the inactivated enzyme solution. The cellulase activity was assayed as described by Bell et al. (1955) with slight modifications. The crude enzyme solution (0.1 mL) was mixed with 0.2 mL of 0.6% carboxymethyl cellulose in 0.05 mol⋅L^–1^ citric acid buffer at pH 4.8 and incubated at 50°C for 30 min. A volume of 1.0 mL of 3, 5-dinitrosalicylic acid (DNS) was immediately added and boiled for 5 min. After cooling, 0.7 mL of deionized water was added, and the absorbance was measured at 540 nm to determine the cellulase activity (μg⋅g^–1^⋅h^–1^). The inactivated enzyme solution was used as the control. The enzyme activity of each treatment was measured three times. Moreover, the activity of pectinase was assayed as described by Bell et al. (1955) with slight modifications. A solution of 0.2 mL of 0.25% polygalacturonic acid in 0.05 mol⋅L^–1^ citric acid buffer at pH 4.8 was added to 0.1 mL of the crude enzyme solution with 0.3 mL citric acid buffer and incubated at 50°C for 1 h. A volume of 1.8 mL of DNS was added, and the mixture was boiled for 5 min. After cooling, the absorbance was measured at 540 nm to determine the pectinase activity (μg⋅g^–1^⋅h^–1^). The inactivated enzyme solution was used as the control. Each treatment was measured once and repeated independently three times.

One gram of the stem was accurately weighed and ground in a mortar with 2 mL of 0.1 mol⋅L^–1^ phosphate buffer (pH 7.8) and a small amount of quartz sand on ice. A volume of 3 mL of the phosphate buffer was added and ground into a homogenate. The sample was centrifuged at 4,000 rpm at 4°C for 15 min, and the precipitate was discarded. The supernatant was collected, and the volume was brought to 10 mL with the phosphate buffer. The solution obtained was used as the crude enzyme solution. A volume of 0.2 mL of the crude enzyme solution was mixed with 0.8 mL of activator (0.1 mol⋅L^–1^ phosphate buffer, pH 7.8, containing 20 mmol⋅L^–1^ Cysteine and 1.0 mmol⋅L^–1^ EDTA) and preheated in a 37°C water bath for 10 min. One mL of 1% casein phosphate buffer (0.1 mol/L, pH = 7.8) was added to this and preheated to 37°C for 10 min. Immediately, 3 mL of trichloroacetic acid (TCA) solution that contained 0.11 mol⋅L^–1^ TCA, 0.22 mol⋅L^–1^ sodium acetate, and 0.33 mol⋅L^–1^ acetic acid was added to this to stop the reaction (control, TCA was added first followed by the substrate casein, incubated stationary for 30 min, and centrifuged at 8,000 rpm for 10 min. The absorbance of the supernatant was measured at 275 nm to determine the protease activity (U⋅g^–1^) (Guo et al., 2006). Each treatment was measured once and repeated independently three times.

Approximately 1 g of stem from one faba bean plant per treatment was ground in a mortar with a small amount of quartz sand and 2 mL of distilled water, and the homogenate was poured into a centrifuge tube with 6 mL of distilled water. The extract was placed at room temperature for 15–20 min and stirred every few minutes for complete extraction. The mixture was centrifuged at 3,000 rpm for 10 min, and the supernatant was brought to a constant volume with distilled water. It was then shaken to obtain the original amylase solution. Ten mL of the original amylase solution was diluted with distilled water to obtain the amylase dilution. Amylase stock solutions (1.0, 1.0, 1.0, 0, 0, and 0 mL) were placed in a water bath at 70°C for 15 min, cooled in running water, mixed with DNS reagent (2, 0, 0, 2, 0, and 0 mL, respectively) and incubated for 10 min in a 40°C water bath. To this, 1.0 mL of 1% starch solution was added and incubated for another 5 min at 40°C. Finally, DNS reagent (0, 2, 2, 0, 2, and 2 mL, respectively) was added to the tubes, shaken, placed in water for 5 min, cooled, and brought to a volume of 20 mL with distilled water. After shaking well, the absorbance was measured at 540 nm to determine the amylase activity (mg⋅g^–1^⋅min^–1^) (Li, 2000). Each treatment was measured once and repeated independently three times.

### Extraction and Measurement of Lignin From Faba Bean Stems

The mercaptoacetic acid method was used as described by Bruce and West (1989). Two grams of one faba plant per treatment of fresh weight of stems were ground in 7 mL of 99.5% ethanol, centrifuged for 30 min at 10,000 *g* at 25°C and then precipitated at room temperature for 12 h. The precipitate was dried, and 50 mg was placed in a centrifuge tube. A volume of 5 mL of 2 N HCl and 0.5 mL of mercaptoacetic acid was steamed in a boiling water bath for 8 h and then cooled in an ice bath. Following centrifugation for 30 min at 10,000 *g* at 4°C, the precipitate was added to 2.5 mL of distilled water and centrifuged at for 5 min at 10,000 *g* and 4°C. The precipitate was suspended in 5 mL of 1 N NaOH and incubated at 25°C for 18 h, during which it was gently stirred several times and then centrifuged at 10,000 *g* for 30 min. The supernatant was removed, and 1 mL of concentrated HCl was added, precipitated at 4°C for 4 h, centrifuged for 30 min at 10,000 *g* to remove the precipitate, and then 3 mL of 1 N NaOH was added to dissolve the precipitate. The relative lignin content (mg⋅g^–1^) was determined at 280 nm with NaOH as a blank control (Bruce and West, 1989). Each treatment was measured once and repeated independently three times.

### Sample Preparation and Observation of the Stem Sections of Faba Bean

Paraffin sections were prepared as described by Wang et al. (2013). The sections of one faba bean plant per treatment were placed in xylene I for 20 min, xylene II for 20 min, anhydrous ethanol I for 5 min, anhydrous ethanol II for 5 min, 75% alcohol for 5 min, rinsed with tap water, and placed in safflower dye for 1–2 h. The sections were washed slightly with tap water to remove the excess dye and decolorized with 50, 70, and 80% gradient alcohol. They were then put in solid green dye for 30–60 s, soaked with anhydrous ethanol in three tanks, and dehydrated. Finally, the slices were transparent in ethanol and xylene for 5 min. The slices were removed from the xylene and dried slightly. After neutral gum sealing, the slices were observed with a microscope (Eclipse CI; Nikon, Tokyo, Japan) (Wang et al., 2013). Four sections (*n* = 4) were made for each treatment and repeated three times, and each section was observed four times.

### Determination of Photosynthetic Physiological Indices in Faba Bean Leaves

A total of 45 days after the faba bean seedlings were transplanted, the leaf gas exchange was measured from selected leaves obtained from the tip of the faba bean stem and harvested between 9:00 and 12:00. The leaves chosen were the second fully expanded leaves of 4–6 fully expanded compound leaves that were not damaged by pathogens. Data of gas exchange parameters, such as the leaf net photosynthetic rate (P*n* μmol CO_2_⋅m^–2^⋅s^–1^), transpiration rate (T*i*, mmol H_2_O⋅m^–2^⋅s^–1^), stomatal conductance (G*s*, mol H_2_O⋅m^–2^⋅s^–1^), and intercellular carbon dioxide concentration (C*i*, μmol⋅mol^–1^) were generated by a LI-6400 Portable Photosynthesis System (LICOR, Lincoln, NE, United States), an open-flow infrared gas analyzer adapted with light and temperature control systems for each leaf sample. The chlorophyll content (SPAD) was determined using an Fk-yl04 chlorophyll meter (Shandong Fangke Instrument Co., Ltd., China). The following conditions were maintained during the gas exchange assay: 25°C air temperature, 80 to 90% relative humidity, 400 μmol⋅mol^–1^ CO_2_ concentration, and 1,000 μmol⋅m^–2^⋅s^–1^. Each treatment was measured once and repeated independently three times.

### Data Processing and Statistical Analysis

SPSS 18.0 (SPSS, Inc., Chicago, IL, United States) was used to statistically analyze the data. Each dataset was tested for homogeneity of variance using a normal probability plot. A multi-factor way analysis of variance (ANOVA) was used to analyze the data. Least significant difference (*LSD*) was used to separate the means between the treatments, which were considered significant at *P* ≤ 0.05. All the data are shown as the mean ± standard error.

## Results

### Effects of FOF and Cinnamic Acid Stress on Faba Bean Wilt and the Intercropping Effect

**FIGURE 2:**
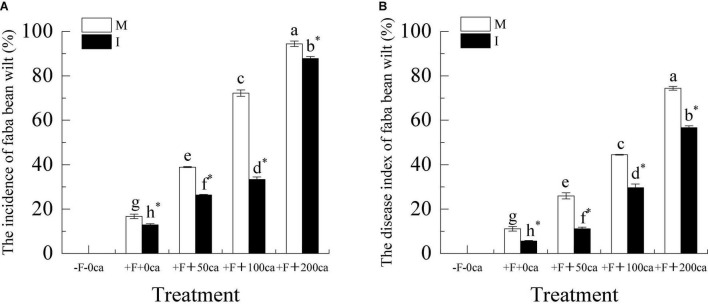
Effects of FOF and cinnamic acid stress on faba bean wilt and the intercropping effect. **(A)** The incidence of faba bean wilt, **(B)** The disease index of faba bean wilt. Values in the figure are the mean ± standard error. Different lowercase letters after the data indicate a significant difference (*P* < 0.05). *Significant differences between monocropping and intercropping treatments with the same FOF and cinnamic acid levels (*P* < 0.05). ca, cinnamic acid; F, Fusarium wilt; FOF, *Fusarium oxysporum* f. sp. *fabae*; I, intercropping; M, monocropping.

As shown in Figure 2 for the faba bean monocropping treatment, compared with the +F+0ca treatment, the treatments of F+50ca, +F+100ca and +F+200ca significantly increased the incidence of faba bean wilt by 133.35, 333.23, and 466.52%, respectively, and significantly increased the faba bean wilt disease index by 133.39, 300, and 570.02%, respectively.

Under the +F+0ca, +F+50ca, F+100ca and +F+200ca treatments, compared with the faba bean monocropping treatment, the treatments of wheat and faba bean intercropping significantly decreased the incidence of faba bean wilt by 22.79, 32.23, 53.84, and 6.9%, respectively, and significantly decreased the faba bean disease index by 49.9, 57.15, 33.32, and 23.87%, respectively.

### Effects of FOF and Cinnamic Acid Stress on the Growth of Faba Bean and Intercropping Effects

**FIGURE 3:**
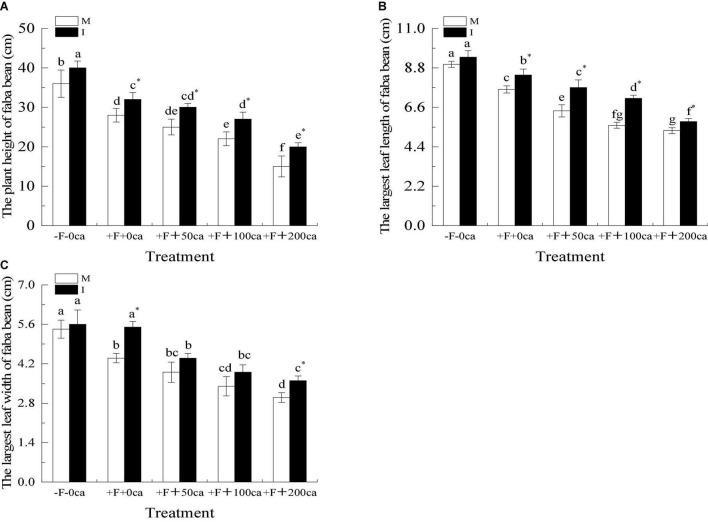
Effects of FOF and cinnamic acid stress on the growth of faba bean (*Vicia faba*) and intercropping effect. **(A)** Plant height of faba bean, **(B)** The largest leaf length of faba bean, **(C)** The largest leaf width of faba bean. Values in this figure are the mean ± standard error. Different lowercase letters after the data indicate significant differences (*P* < 0.05). *significant differences between monocropping and intercropping treatments with the same FOF and cinnamic acid levels (*P* < 0.05). ca, cinnamic acid, FOF, *Fusarium oxysporum* f. sp. *fabae*; I, intercropping; M, monocropping.

As shown in Figure 3, under the faba bean monocropping treatment compared with the -F-0ca treatment, the treatment of +F+0ca significantly decreased the plant height, maximum leaf length and maximum leaf width by 22, 16, and 19%, respectively,

Under the faba bean monocropping treatment, compared with the +F+0ca treatment, the treatment of +F+50ca resulted in a significant decrease in the maximum leaf length of faba bean by 16%. Compared with the +F+0ca treatment, the treatments +F+100ca and +F+200ca significantly decreased the plant height, maximum leaf length and maximum leaf width by 21 and 46, 26 and 30%, and 23 and 32%, respectively.

Under the +F+0ca, +F+ 50ca, F+100ca and +F+200ca treatments, compared with the faba bean monocropping treatment, the treatments of wheat and faba bean intercropping significantly increased the plant height and maximum leaf length by 14, 20, 23, and 33%, respectively, and 11, 20, 27, and 9%, respectively. Under the +F+0ca and +F+ 200ca treatments, compared with the faba bean monocropping treatment, the treatments of wheat and faba bean intercropping significantly increased the maximum leaf width by 25 and 20%, respectively.

### Effects of FOF and Cinnamic Acid Stress on the Cell Wall Degrading Enzyme Activity of Stem of Faba Bean and Intercropping Effects

**FIGURE 4:**
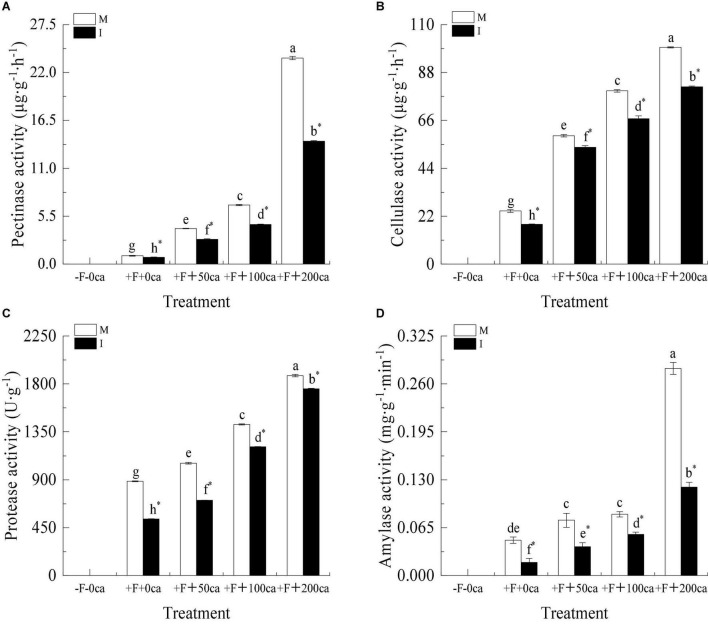
Effects of FOF and cinnamic acid stress on stem cell wall degradation enzyme of faba bean and intercropping effect. **(A)** Pectinase, **(B)** cellulase, **(C)** protease, **(D)** amylase. Values in the figure are the mean ± standard error. Different lowercase letters after data indicate significant difference (*P* < 0.05). *Significant differences between monocropping and intercropping treatments with the same FOF and cinnamic acid levels (*P* < 0.05). ca, cinnamic acid; FOF, *Fusarium oxysporum* f. sp. *faba*; I, intercropping; M, monocropping.

As shown in Figure 4, under the faba bean monocropping treatment, compared with the +F+0ca treatment, the treatments of F+50ca, +F+100ca and +F+200ca significantly increased the activities of pectinase in the faba bean stems by 325, 605.89, and 2,364.92%, respectively, significantly increased the activities of cellulase in the faba bean stems by 142, 226.57, and 308.64%, respectively, significantly increased the activities of protease in the faba bean stems by 19.06, 60.07, and 111.93%, respectively, and significantly increased the activities of amylase in the faba bean stems by 56.25, 72.91, and 485.41%, respectively.

Under the +F+0ca, +F+50ca, F+ 100ca and +F+200ca treatments, compared with the faba bean monocropping treatment, the treatments of wheat and faba bean intercropping significantly decreased the activities of pectinase in the faba bean stems by 19.44, 30.47, 33.15, and 40.34%, respectively, significantly decreased the activities of cellulase in the faba bean stems by 25.07, 8.94, 16.05, and 18.26%, respectively, significantly decreased the activities of protease in the faba bean stems by 40.12, 32.96, 14.67, and 6.58%, respectively, and significantly decreased the activities of amylase in the faba bean stems by 62.5, 48, 32.53, and 57.29%, respectively.

### Effects of FOF and Cinnamic Acid Stress on Lignin in Faba Bean Stems and the Intercropping Effects

**FIGURE 5:**
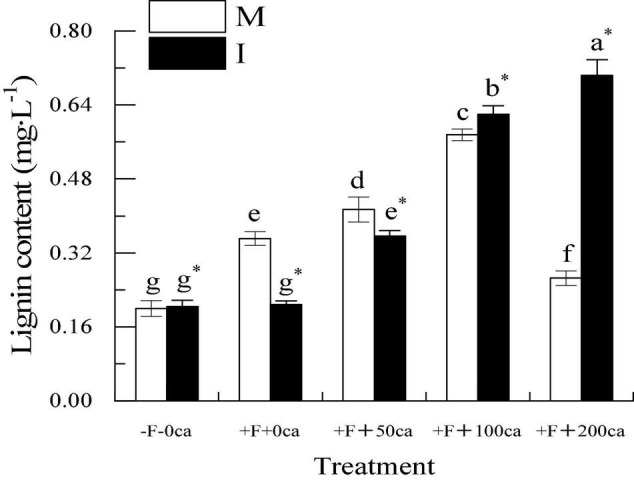
Effects of FOF and cinnamic acid stress on lignin in the stems of faba bean (*Vicia faba*) and the effect of intercropping. Values in the figure are the mean ± standard error. Different lowercase letters after data indicate significant difference (*P* < 0.05). *Significant differences between monocropping and intercropping treatments at the same FOF and cinnamic acid levels (*P* < 0.05). ca, cinnamic acid; FOF, *Fusarium oxysporum* f. sp. *fabae*; I, intercropped; M, monocropped.

As shown in Figure 5, under the faba bean monocropping treatment, compared with the -F-0ca treatment, the treatment of +F+0ca significantly increased the lignin content of faba bean by 75.5%. Compared with the +F+0ca treatment, the treatments of +F+50ca and +F+100ca significantly increased the lignin content in the faba bean stems by 17.94 and 63.98%, respectively. Compared with the +F+0ca treatment, the treatment of +F+200ca significantly decreased the lignin content in the faba bean stems by 24.41%.

Under the +F+0ca and +F+50ca treatments, compared with the faba bean monocropping treatment, the treatments of wheat and faba bean intercropping significantly decreased the contents of lignin in the faba bean stems by 40.74 and 13.86%, respectively. Under the F+100ca and +F+ 200ca treatments, compared with the faba bean monoculture treatment, the treatments of wheat and faba bean intercropping significantly increased the lignin content in the faba bean stems by 7.71 and 165.35%, respectively. The results showed that in wheat and faba bean intercropping, the lignin content in stem of faba bean decreased significantly under the treatments of FOF and FOF with low concentrations of cinnamic acid after FOF inoculation.

### Effects of FOF and Cinnamic Acid Stress on Tissue Structure in the Stems of Faba and Intercropping Effects

**FIGURE 6:**
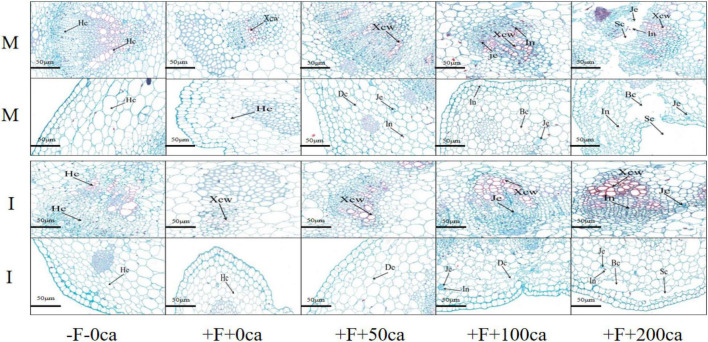
Effects of FOF and cinnamic acid stress on tissue structure in the faba bean (*Vicia faba*) stems and the intercropping effect. Hc: Healthy cell, Xcw: Xylem cell wall, Je: Jelly cell, In: Inclusion, Dc: Deformed cell, Bc: Broken cell, Sc: Schismatic cell layer.

As shown in Figure 6, the production of paraffin sections enabled microscopic observation that showed that under faba bean monocropping treatment, compared with the -F-0ca treatment, the tissue structure of stems following treatment with +F+0ca was closely arranged and intact. However, the cell wall of conduit tissue was thickened. Compared with the +F+0ca treatment, the +F+50ca treatment resulted in stem duct tissue cells that were more thickened, and small amounts of gelatinous substances and inclusions appeared in the basic tissue cells of the cortex, and the cells twisted. Under the faba bean monocropping treatment, compared with the +F+0ca treatment, the +F+100ca treatment resulted in thickening in the ductal tissue cells of the stem, and the cells of cambium tissue showed colloid substances and inclusions. The basic tissue cells displayed colloid substances; inclusion of the cortex further increased, and the basic tissue cells of cortex partially broke. Under the faba bean monocropping treatment, compared with the +F+0ca treatment, in the +F+200ca treatment, the conduit tissue thickened and decreased; the cambium cells displayed many gelatinous substances and inclusions, and some cambium cells showed the phenomenon of a dividing cell layer with a hollow cavity. Many gelatinous substances and inclusions appeared in the basic tissue cells of the cortex, and many of the basic tissue cells of the cortex showed the phenomenon of broken cells and a cavity dividing cell layer. In addition, many tissue cells had died. The results showed that the faba beans after FOF inoculation added different concentrations of cinnamic acid, which thickened the catheter tissue stem cells, cambium tissue cells in colloidal material, inclusions and splinter cell layers in the cavity, cortex tissue cells in the colloidal material, basic contents and cell disruption and cavity splinter cell layer.

Under the -F-0ca treatment, compared with the faba bean monocropping treatment, there was no difference in the structures of stem tissues and cells between the wheat and faba bean intercropping treatments. Under the +F+0ca treatment, compared with the faba bean monocropping treatment, the wheat and faba bean intercropping treatments led to a lower degree of thickening of the conduit tissue cells that was less than that in the faba bean monocropping treatment. Under the +F+50ca treatment, compared with the faba bean monocropping treatment, the wheat and faba bean intercropping treatments resulted in additional thickening of the ductal tissue cells of faba bean stems, and the basic tissue cells of cortex were only distorted. No gelatinous substances and inclusions were found. Under the +F+100ca treatment, compared with the faba bean monocropping treatment, there were no inclusions in the cambium tissue cells and many cell contortions in the basic tissue cells of cortex of faba bean following the wheat and faba bean intercropping treatments. Under the F+200ca treatment, compared with the faba bean monocropping treatment, the wheat and faba bean intercropping treatments significantly thickened the ductal tissue cells in the faba bean stems, and the cambium tissue cells did not exhibit the phenomenon of dividing cell layers in the cavity, while the basic tissue cells in the cortex only had a small amount of cell fragmentation and dividing cell layer cavity. The results showed that wheat and faba bean intercropping effectively increased the integrity of stem tissue structure.

### Effects of FOF and Cinnamic Acid Stress on the Photosynthetic Physiology of Faba Bean Leaves and the Intercropping Effects

**FIGURE 7:**
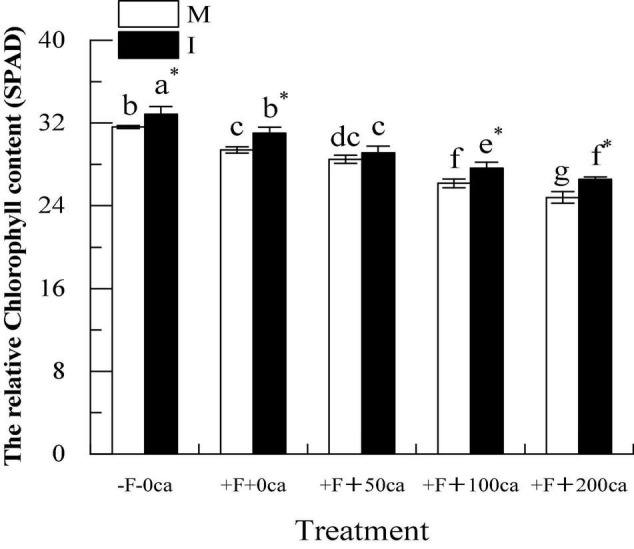
Effects of FOF and cinnamic acid stress on the relative chlorophyll content of faba bean leaves and the intercropping effect. Values in the table are mean ± standard error. Different lowercase letters after data indicate significant difference (*P* < 0.05). *Significant differences between monocropping and intercropping treatments with the same FOF and cinnamic acid levels (*P* < 0.05). ca, cinnamic acid; FOF, *Fusarium oxysporum* f. sp. *fabae*; I, intercropping: M, monocropping.

As shown in Figure 7, under the faba bean monocropping treatment, compared with the -F-0ca treatment, the treatment of +F+0ca significantly decreased the relative chlorophyll content of faba bean leaves by 7.05%.

Under the faba bean monocropping treatment, compared with +F+0ca, the treatment of +F+50ca did not significantly change the relative chlorophyll content of the faba bean leaves. However, treatments with +F+100ca and +F+200ca significantly decreased the relative chlorophyll content of faba bean leaves by 10.99 and 15.64%.

Under the -F-0ca, +F+0ca, +F+100ca and F+200ca treatments, compared with the faba bean monocropping treatment, the treatments of wheat and faba bean intercropping significantly increased the relative chlorophyll content of the faba bean leaves by 3.89, 5.55, 5.60, and 7.12%, respectively. The results showed that wheat and faba bean intercropping could significantly increase the relative chlorophyll content of faba bean leaves.

**FIGURE 8:**
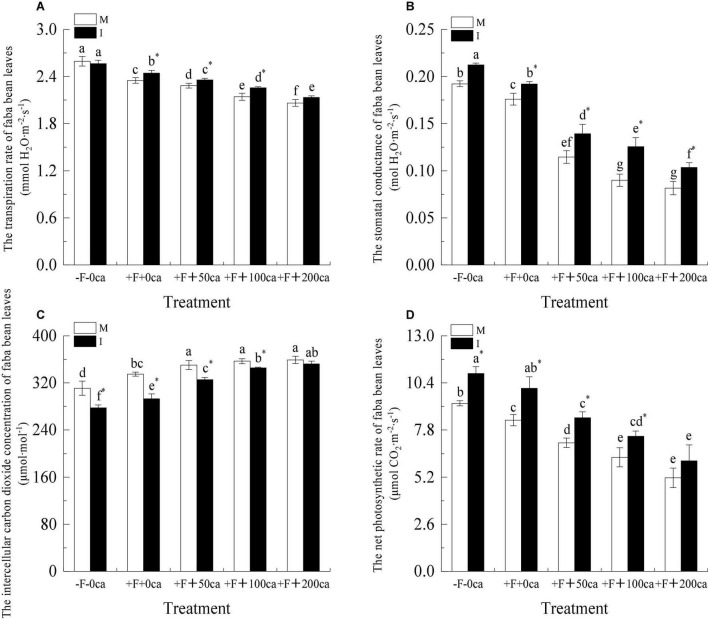
Effects of FOF and cinnamic acid stress on the photosynthesis of faba bean (*Vicia faba*) leaves and intercropping effect. **(A)** Transpiration rate (T*i*), **(B)** Stomatal conductance (G*s*), **(C)** Intercellular carbon dioxide concentration (C*i*), **(D)** Net photosynthetic rate (P*n*). Values in the table are mean ± standard error. Different lowercase letters after data indicate significant differences (*P* < 0.05). *Significant differences between monocropping and intercropping treatments with the same FOF and cinnamic acid levels (*P* < 0.05). ca, cinnamic acid; FOF, *Fusarium oxysporum* f. sp. *pisi*; I, intercropping; M, monocropping.

As shown in Figure 8, under the faba bean monocropping treatment, compared with -F-0ca, the treatment of +F+0ca significantly decreased the T*i*, G*s* and P*n* of faba bean leaves by 9.38, 8.47, and 10.01%, respectively, and significantly increased the C*i* of faba bean leaves by 7.68%.

Under faba bean monocropping treatment, compared with the +F+0ca treatment, the treatments of +F+ 50ca, +F+ 100ca and +F+ 200ca significantly decreased the T*i* of faba bean leaves by 2.83, 8.79, and 12.2%, respectively, significantly decreased the G*s* of faba bean leaves by 34.88, 48.86, and 53.63%, respectively, significantly decreased the P*n* of faba bean leaves by 15, 24.49, and 38.12%, respectively, and significantly increased the C*i* of faba bean leaves by 4.61, 6.57, and 7.18%, respectively.

Under the -F-0ca treatment, compared with the faba bean monocropping treatment, the treatments of wheat and faba bean intercropping significantly decreased the C*i* of faba bean by 10.72% and increased the P*n* of faba bean by 17.76%. Under the +F+0ca, +F+50ca and F+100ca treatments, compared with the faba bean monocropping treatment, the treatments of wheat and faba bean intercropping significantly increased the T*i* of faba bean leaves by 3.97, 3.21, and 5.28%, respectively, significantly increased the G*s* of faba bean leaves by 9.09, 21.55, and 3.96%, respectively, significantly increased the P*n* of faba bean leaves by 21.11, 19.57, and 18.27%, respectively, and significantly decreased the C*i* of faba bean leaves by 12.49, 7.13, and 3.2%, respectively. Under the F+200ca treatment, compared with the faba bean monocropping treatment, the treatments of wheat and faba bean intercropping of faba bean leaves significantly increased the G*s* by 27.16%.

## Discussion

In recent years, an increasing amount of attention has been paid to cinnamic acid, which plays a key role in promoting Fusarium wilt (Li et al., 2014a; Zhang et al., 2020). Research has shown that compared with the treatment that lacked cinnamic acid, exogenous treatment with cinnamic acid promoted the incidence of cucumber wilt by 214.4–266.8% (Ye et al., 2004). Tian et al. (2019) found that the exogenous addition of ferulic acid, a derivative of cinnamic acid secreted by strawberry (*Fragaria* × *ananassa*) roots, could improve the disease index of strawberry wilt by 37.03%. In this study, we obtained similar results to the study above. Under the faba bean monocropping treatment, compared with the +F+0ca treatment, the +F+50ca, +F+ 100ca and +F+200ca treatments significantly increased the incidence and disease index of faba bean wilt (Figure 2). These results indicated that cinnamic acid plays an important role in promoting the occurrence of faba bean wilt. Some studies have shown that reasonable intercropping is an effective measure for disease control. Compared with watermelon monocropping, wheat and watermelon intercropping significantly reduced the incidence of watermelon wilt (Xu et al., 2015). Studies have also shown that compared with the tomato monocropping treatment, tillering onion and tomato intercropping treatment can effectively reduce the incidence and disease index of Verticillium wilt in tomato by 35.58 and 19.83%, respectively (Fu et al., 2015). In this study, we obtained similar results to the previous study above. Under the +F+0ca, +F+50ca, +F+100ca and +F+200ca treatments, a comparison of the faba bean monocropping treatment with the wheat and faba bean intercropping treatments significantly decreased the incidence and disease index of faba bean wilt (Figure 2). The results showed that wheat and faba bean intercropping could effectively control the occurrence of faba bean wilt.

Allelopathic autotoxic substances can not only promote the occurrence of soilborne diseases but also directly inhibit the normal growth and development of crops. Studies have shown that compared with a lack of cinnamic acid, treatment with exogenous cinnamic acid can significantly reduce the aboveground and underground dry weight and the leaf area growth rate of cucumber plants (Li et al., 2017). In this study, we obtained results similar to the study by Li et al. (2017). Under the faba bean monocropping treatment, compared with the -F-0ca treatment, the +F+0ca treatment significantly reduced the plant height, maximum leaf width and maximum leaf length of faba bean. Compared with the +F+0ca treatment, the +F+50ca, +F+100ca and +F+200ca treatments significantly reduced the plant height, maximum leaf width and maximum leaf length of faba bean even more (Figure 3). The results showed that cinnamic acid inhibited the growth of faba bean shoots. Studies have shown that intercropping can promote plant growth. Compared with cucumber monocropping, garlic and cucumber intercropping significantly increased the shoot and root biomass of cucumber (Xiao et al., 2013). In this study, we obtained results that were similar to those of Xiao et al. (2013). Under the +F+0ca treatment, the +F+ 50ca, +F+ 100ca and +F+ 200ca treatments, compared with faba bean monocropping treatment, the wheat and faba bean intercropping treatments significantly increased the plant height, maximum leaf length and maximum leaf width of faba bean (Figure 3). The results showed that wheat and faba bean intercropping could alleviate the synergistic effect of FOF and cinnamic acid and promote the growth of faba bean.

When host plants are infected by pathogens, CWDEs produced by plant pathogenic fungi are considered to be important pathogenic factors (Annis and Goodwin, 1997). Gharbi et al. (2015) found that the CWDEs secreted by *Verticillium dahliae* were closely related to the occurrence of *V. dahliae* in olive (*Olea europaea*), potato and sunflower (*Helianthus annuus*). Studies have shown that allelopathic autotoxic substances can improve the activity of pathogenic factors (CWDEs) secreted by pathogens. Wu et al. (2008b) found that the activities of pectinase (590%), cellulase (760%), amylase (2006%) and protease (27.0%) produced by *F. oxysporum* increased significantly after cinnamic acid was added to the culture medium of this fungus (Wu et al., 2008b). In this study, we obtained results similar to those of Wu et al. (2008b). Under the faba bean monocropping treatment, compared with the +F+0ca treatment, the +F+50ca, +F+100ca and +F+ 200ca treatments significantly increased the activities of pectinase, cellulase, protease and amylase produced by FOF in the stems of faba bean (Figure 4). The results showed that cinnamic acid promoted FOF to produce more active CWDEs in faba bean, improved its pathogenicity, and created favorable conditions for the further infection of faba bean. Studies have shown that different CWDEs require synergistic action during the pathogenic process of pathogens to effectively improve the disease risk of the host (Guo et al., 2019). In the pathogenic process of *Xanthomonas* on rice, different CWDEs need to be secreted to act synergistically and promote the occurrence of rice wilt (Ray et al., 2000). In this study, we concluded that FOF synergistically causes disease to faba beans by secreting cellulase, pectinase, protease and amylase and may cause more damage to faba beans. In this study, under the +F+0ca, +F+50ca, +F+100ca and +F+200ca treatments, compared with the faba bean monocropping treatment, the wheat and faba bean intercropping treatments significantly reduced the activities of pectinase, cellulase, protease and amylase in faba bean stems (Figure 4). This is similar to the results of Li C. X. (2019), who showed that the root exudates of wheat could reduce the activities of pectinase, cellulase, protease and amylase produced by *F. oxysporum* (Li C. X., 2019). The results obtained in this study could be because the wheat root exudates in the wheat and faba bean intercropping system reduced the activity of CWDEs produced by FOF in faba bean. The results showed that wheat and faba bean intercropping could reduce the virulence of FOF to faba bean by decreasing the activity of CWDEs produced by FOF in faba bean.

To successfully infect plants, pathogens must overcome the mechanism of host resistance formed during coevolution. The resistance of tissue structure is the first host defense of pathogens. This primarily refers to some components of the cell wall, stomatal special structure, small molecule resistant substances, proteins that destroy fungal cell permeability, and ribosome inactivation proteins among others. The resistance to tissue structure is related to the contents of cutin, lignin and lignin, and the changes of these components directly affect the innate resistance of plants. Lignin is a complex polymer that is found in the secondary cell wall of plants. It plays a crucial role in the solidification of cell walls and creates a non-degradable barrier for pathogens, thus, strengthening the protection of plants against biological stress (Bonawitz and Chapple, 2010; Moura et al., 2010). In this study, under the faba bean monocropping treatment, compared with the -F-0ca treatment, treatment with +F+0ca significantly increased the lignin content in faba bean stems (Figure 5). The possible reason is that the stress response of faba beans was activated following FOF inoculation. The +F+0ca, +F+50ca, +F+100ca and +F+200ca treatments showed a “low promoting and high inhibiting” effect on lignin content in the faba bean stems (Figure 5). Under the faba bean monocropping treatment, compared with the +F+0ca, the +F+50ca and +F+100ca treatments further increased the lignin content in faba bean stems (Figure 5). The possible reason was that the stress response of faba bean was further activated under 50 mg⋅L^–1^ and 100 mg⋅L^–1^ cinnamic acid stress, which promoted the increase in lignin synthesis. Under the faba bean monocropping treatment, compared with +F+0ca, the +F+ 200ca treatment significantly decreased the lignin content in faba bean stems (Figure 5). The possible reason is that when the concentration of cinnamic acid reaches 200 mg⋅L^–1^, the resistance of the tissue structure of faba bean is not enough to resist the damage of cinnamic acid, and the cell wall of the tissue defense mechanism cannot be thickened, thus, reducing the resistance of faba bean to FOF infection. These results indicate that cinnamic acid could inhibit lignin synthesis in faba bean stems and reduce resistance to FOF, which could be an important mechanism by which cinnamic acid promotes the occurrence of faba bean wilt. In this study, under the +F+0ca and +F+50ca treatments, compared with the faba bean monocropping treatment, the wheat and faba bean intercropping treatments significantly reduced the lignin content in the faba bean stems (Figure 5). Studies have shown that the root exudates of wheat can inhibit the activity of *F. oxysporum* that causes Fusarium wilt in watermelon (Lv et al., 2018). In this study, the probable cause was that wheat root exudates inhibited the activity of FOF in wheat and faba bean intercropping. Under the +F+100ca and +F+200ca treatments, compared with the faba bean monocropping treatment, the wheat and faba bean intercropping treatments significantly increased the lignin content in faba bean stems (Figure 5). In this study, the possible reason was that 100 mg⋅L^–1^ and 200 mg⋅L^–1^ of cinnamic acid promoted the pathogenicity (CWDEs) of FOF, and the stress response of faba beans was not enough to resist the damage of FOF. Studies have shown that the root exudates of wheat can increase the content of lignin in watermelon (Li C. X., 2019). Wheat helped the faba bean to activate the resistance of tissue structure by exuding root exudates, and lignin synthesis increased substantially. The results showed that wheat and faba bean intercropping could reduce the damage of FOF and improve the resistance of tissue structure of faba bean. This could be an important mechanism for the effective control of faba bean wilt in wheat intercropping.

By observing the cell structures of plant tissue, we can study the changes in cell structure of plant tissue under stress, which can provide a cytological basis for plant injury. Studies have shown that pathogen invasion can promote the thickening of potato cell walls (Perry and Evert, 1983). In this study, we observed paraffin sections of plant tissue cells and found that under the faba bean monocropping treatment, compared with the -F-0ca treatment, the +F+0ca treatment resulted in a thickening of the xylem vessels of faba bean stems (Figure 6). This could be the stress response of faba bean to FOF under FOF stress. Further studies have shown that allelopathic autotoxic substances can destroy the tissue and cell structure of plants. Qi et al. (2015) found that under the stress of *p*-hydroxybenzoic acid, cells in the epidermis, subcutaneous and middle column of the strawberry root system, resulting in severe damage (Qi et al., 2015). In this study, under the faba bean monocropping treatment, compared with the +F+0ca treatment, the xylem vessels in faba bean stems became even thicker in the +F+50ca, +F+ 100ca and +F+ 200ca treatments, and the basic tissue cells of the cambium and cortex were invaded by gelatinous substances and inclusions (Figure 6). The degree of cell distortion, cell fragmentation, cell structure dispersion and even cell cavities was aggravated. The possible reason is that cinnamic acid promotes the production of high levels of CWDE activity by FOF, which leads to the leakage of a large amount of cell structures and lignin from the stem, and the breakdown of cell defense system (Figure 6). These results indicate that cinnamic acid can promote the pathogenicity of FOF, further aggravating the damage to stem tissue and cell structures, which could be one of the important mechanisms that enables cinnamic acid to promote the occurrence of faba bean wilt. In this study, under the +F+0ca, +F+ 50ca, +F+100ca and +F+200ca treatments, compared with the faba bean monocropping treatment, the wheat and faba bean intercropping treatments cause a reduction in the amounts of gelatinous substances and inclusions in the tissue structure of faba bean stems; fewer cells were twisted and broken, and the degree of cavities in the divided cambium was reduced (Figure 6). Studies have shown that the root exudates of wheat can reduce spore germination, sporulation and mycelial growth of *F. oxysporum* f. sp. *niveum*, the causal agent of watermelon Fusarium wilt (Lv et al., 2018). In this study, we hypothesized that the root exudates of wheat in the wheat and faba bean intercropping inhibited the growth and reproduction of FOF and reduced the damage of FOF to faba bean.

After pathogens invade, they can cause the chlorosis of leaves and reduce the photosynthetic physiological characteristics of plants and promote the occurrence of diseases (Kim et al., 2010; Xie et al., 2020). Chlorophyll is the main pigment in plant photosynthesis, which can absorb, transfer and transform light energy. The level of pigment content in plant leaves directly affects the strength of plant photosynthesis (Mohsenzadeh et al., 2006). Gamliel et al. (1997) showed that the chlorophyll (21.19%) content decreased after potato was infected with Verticillium wilt. In this study, under the faba bean monocropping treatment, compared with the -F-0ca treatment, the +F+0ca treatment significantly reduced the relative chlorophyll phase content of faba bean leaves (Figure 7). Studies have shown that autotoxic substances can reduce the content of chlorophyll in plant leaves. Baziramakenga et al. (1994) found that the addition of cinnamic acid to soybean reduced the chlorophyll (27%) content in soybean leaves compared with the control. In this study, we obtained similar results. Under the faba bean monocropping treatment, compared with the +F+0ca treatment, the +F+50ca, +F+100ca and +F+200ca treatments significantly reduced the relative chlorophyll content of faba bean leaves (Figure 7). Studies have shown that compared with the treatment without *F. oxysporum* inoculation, the treatment with *F. oxysporum* f. sp. *cucumerinum* decreased the P*n* and G*s* of cucumber leaves and increased the C*i* (Ye et al., 2004). Studies have also shown that allelopathic autotoxicity can reduce plant photosynthesis and promote disease occurrence. Compared with the lack of cinnamic acid addition, the cinnamic acid treatment reduced the P*n* and G*s* of cucumber leaves, increased the C*i*, and promoted the occurrence of Fusarium wilt (Ye et al., 2004). In this study, we obtained similar results to those described above under the faba bean monocropping treatment, which compared with -F-0ca, the +F+0ca treatment, significantly reduced the T*r*, G*s* and P*n* of faba bean leaves but significantly increased the C*i* (Figure 8). Compared with the +F+0ca treatment, the +F+50ca, +F+100ca and +F+200ca treatments further significantly reduced the T*r*, G*s* and T*r* but significantly increased the Ci (Figure 8). The possible reason is that the factors that lead to the decrease in photosynthetic rate under adverse conditions primarily include stomatal and non-stomatal factors. Whether stomatal or non-stomatal factors are the primary reasons for the decrease in P*n* can be determined by changes in the G*s* and C*i* (Farquhar and Sharkey, 1982). If the G*s* decreases under stress, the C*i* should clearly decrease, and the P*n* should decrease. The change in direction of change in the C*i* and P*n* should be the same. The primary reason for the decrease in P*n* was the decrease in stomatal conductance. If the G*s* decreased while the C*i* remained unchanged or even increased, then the decrease in photosynthetic rate should be caused by non-stomatal factors, such as a reduction in the ability of mesophyll cells to assimilate compounds (Hartley et al., 2006). The direct cause of this non-stomatal factor could be the destruction of the chloroplasts of faba bean leaves, which resulted in a reduction in chlorophyll synthesis and a loss of the ability to assimilate CO_2_. Finally, the photosynthetic physiology of faba bean decreases, caused the wilting phenomenon of faba bean leaves and causing the leaves to turn yellow. In this study, under the +F+0ca, +F+100ca and +F+200ca treatments, compared with the faba bean monocropping treatment, the wheat and faba bean intercropping treatments significantly increased the relative percentage content of faba bean leaves that are green (Figure 7). Under the +F+0ca, +F+50ca and +F+ 100ca treatments, compared with the faba bean monocropping treatment, the wheat and faba bean intercropping treatments significantly increased the T*i*, G*s*, and P*n* and decreased the C*i* (Figure 8). Studies have shown that the root exudates of wheat can inhibit the activities of pathogenic bacteria (Lv et al., 2018). In this study, we hypothesized that the possible reason was that the wheat root secretion in wheat and faba bean intercropping inhibited the activity of FOF, indirectly improving the ability of faba beans to defend themselves, reducing the energy requirement for faba beans to defend themselves, reducing the respiration of faba bean, and then reducing the intercellular carbon dioxide concentration in faba bean leaves. However, under the +F+200ca treatment, compared with the faba bean monocropping treatment, the wheat and faba bean intercropping treatments did not significantly change the T*i*, C*i* and P*n* of faba bean leaves (Figure 8). The possible reason is that the effect of wheat and faba bean intercropping could be limited. As we observed in a previous study, the tissue structure of stems was severely damaged under the dual stress of FOF and 200 mg⋅L^–1^ cinnamic acid. We hypothesized that the tissue and cell structure of faba bean leaves could be seriously damaged under the double stress of FOF and 200 mg⋅L^–1^ cinnamic acid, and the normal photosynthetic function would be lost. The results showed that wheat and faba bean intercropping could significantly increase photosynthesis and decrease the occurrence of faba bean wilt under a particular range of FOF and cinnamic acid stress.

The combined action of autotoxic substances and soilborne pathogens leads to the occurrence of serious soilborne diseases and the inhibition of plant growth in recent years (Li Y. et al., 2019). In our study, the occurrence of faba bean wilt was explained by examining the increase in the *in vivo* activity of the CWDEs of FOF and the reduction in the tissue resistance of faba bean. This shows that the occurrence of the faba bean wilt is a complex process. Wheat and faba bean can reduce the *in vivo* activity of CWDEs in FOF to improve the tissue resistance of faba bean and reduce the occurrence of wilt. We planted a resistant faba bean variety (“89–147”) in soil where faba beans have been continuously cultivated for many years and found that faba bean wilt occurred. The incidence of faba bean wilt was reduced through the use of wheat and faba bean intercropping (Supplementary Data). The possible reason for this is that, in actual field production, the occurrence of faba bean wilt is owing to multiple factors. On the one hand, it could be that FOF can survive in continuous soil for many years. Alternatively, with the increase of its continuous cropping years, faba bean secretes autotoxic substances in the rhizosphere soil that continuously accumulate and aggravate the rhizosphere soil habitat. It works in concert with FOF. Moreover, the resistance of the host decreased. In the future, how to prevent the faba bean wilt caused by multiple factors should not be initiated from the perspective of a single control of pathogenic fungi but should be considered through a comprehensive control strategy. Therefore, we used disease-resistant varieties to improve the resistance of our hosts and improve the microecological environment of the rhizosphere by combining diversified planting (intercropping) to inhibit the growth of pathogenic fungi. A new control model of faba bean Fusarium wilt disease was developed from the combination of host resistance, rhizosphere microecology and pathogenic fungal interaction. The further application of this model will play an important role in sustainably and effectively controlling the occurrence and harm of faba bean wilt, protecting the ecological environment, improving the photosynthetic ability of faba bean, promoting the quality of faba bean products and increasing the income of farmers.

## Conclusion

Cinnamic acid increased the activity of CWDEs secreted by FOF in the stems, reduced the resistance of tissue and cell structure of faba bean, created favorable conditions for FOF to infect faba bean, reduced photosynthesis in the leaves, and promoted the occurrence of faba bean wilt. Wheat and faba bean intercropping decreased the activity of CWDEs secreted by FOF in the stem and improved the resistance of tissue structure of faba bean, thus, enhancing the leaf photosynthesis of faba bean and reducing the occurrence of faba bean wilt.

## Data Availability Statement

The raw data supporting the conclusions of this article will be made available by the authors, without undue reservation.

## Author Contributions

WY conceived the original screening and research plans, designed the experiments and analyzed the data, and finished writing this thesis. YG assisted in the design of the experiment and proposed some suggestions for modification of this manuscript to WY. YL assisted in the data analysis to WY. YZ provided the technical assistance to WY. YD and KD supervised the experiments, agreed to serve as the author responsible for contact and ensures communication. All authors contributed to the article and approved the submitted version.

## Conflict of Interest

The authors declare that the research was conducted in the absence of any commercial or financial relationships that could be construed as a potential conflict of interest.

## Publisher’s Note

All claims expressed in this article are solely those of the authors and do not necessarily represent those of their affiliated organizations, or those of the publisher, the editors and the reviewers. Any product that may be evaluated in this article, or claim that may be made by its manufacturer, is not guaranteed or endorsed by the publisher.
